# Effective doses of propofol and oliceridine fumarate for painless hysteroscopy: a two-phase dose-finding study using Dixon’s up-and-down method

**DOI:** 10.3389/fphar.2026.1945339

**Published:** 2026-10-14

**Authors:** Jinming Chen, Yunhao Shao, Hao Sun, Zhongqi Zhang

**Affiliations:** Department of Anesthesiology, The Affiliated Shunde Hospital of Jinan University, Foshan, China

**Keywords:** Dixon’s up-and-down method, effective dose, oliceridine, painless hysteroscopy, propofol

## Abstract

**Background:**

Painless hysteroscopy is widely performed in gynecological practice, yet achieving an optimal balance between anesthetic safety and patient comfort remains challenging. Combining propofol with oliceridine may mitigate respiratory depression and injection pain associated with conventional regimens, though the effective doses for this combination have not been established. This study sought to identify the optimal dose combination of propofol and oliceridine for use in painless hysteroscopy.

**Methods:**

Fifty-one women scheduled for elective painless hysteroscopy were enrolled in this prospective, two-phase dose-finding study. In Phase I, all patients received a fixed 1 mg dose of oliceridine at induction; propofol was subsequently titrated from an initial 2 mg/kg using Dixon’s up-and-down sequential method in 0.1 mg/kg intervals. Probit regression modeling was used to derive propofol ED50 and ED95, with 95% confidence intervals (CIs) obtained via the delta method; isotonic regression served as sensitivity analysis. In Phase II, propofol was fixed at the Phase I ED50, and oliceridine was titrated from 1 mg in 0.1 mg increments, with ED50 and ED95 determined identically. An effective response was defined as no body movement, no frowning, and a Ramsay Sedation Score ≥5 during cervical dilation and hysteroscope insertion. Secondary outcomes included Nociception Level Index (NOX), Cerebral State Index (CSI), hemodynamic parameters, and adverse events.

**Results:**

The ED50 and ED95 of propofol were 1.818 mg/kg (95% CI: 1.696–1.940) and 2.223 mg/kg (95% CI: 1.840–2.605), respectively. For oliceridine, the corresponding ED50 was 0.663 mg (95% CI: 0.605–0.720) and ED95 was 0.813 mg (95% CI: 0.692–0.934). Pearson goodness-of-fit tests confirmed adequate model fit for both phases (propofol: P = 0.755; oliceridine: P = 0.899), and isotonic regression yielded ED50 estimates within 3% of probit-derived values. Hemodynamic parameters remained stable throughout sedation, with no serious adverse events observed.

**Conclusion:**

The propofol-oliceridine appears to be a feasible option for painless hysteroscopy, with an acceptable safety profile provided that continuous airway monitoring is maintained. The ED50 and ED95 values observed in this study offer preliminary dosing guidance for outpatient practice.

**Clinical Trial Registration:**

www.chictr.org.cn/showproj.html?proj=241260, identifier ChiCTR2500102118.

## 1 Introduction

Despite its essential role in gynecologic diagnosis and therapy, hysteroscopy is associated with moderate to severe pain in 30%–60% of patients undergoing the procedure (Paulo et al., 2016; Guraslan et al., 2022). Propofol-opioid combinations are the mainstay of sedation for this procedure, but respiratory depression, hypotension, and postoperative nausea remain significant concerns (Yu et al., 2019; Ryu et al., 2008; Ma et al., 2025). Furthermore, injection pain occurs in approximately 60% of patients receiving propofol monotherapy (Song et al., 2004; Jalota et al., 2011), underscoring the necessity for enhanced anesthetic approaches.

Oliceridine (Olinvyk™), developed by Trevena, Inc., is a novel μ-opioid receptor biased agonist with δ-opioid receptor antagonist properties, formulated as the fumarate salt. It provides analgesic efficacy comparable to morphine while producing fewer typical opioid-related adverse effects (Goudra, 2022; Ma et al., 2025; Bergese et al., 2020; Bergese et al., 2019). Clinical studies report low rates of respiratory depression (0.4%) and nausea/vomiting (≤4.4%) that are independent of BMI (Soergel et al., 2014b). The drug’s rapid onset (2–5 min), short elimination half-life (1.3–3.7 h), and dual hepatic/renal clearance make it well suited for procedural sedation. Recent findings indicate that the propofol–oliceridine combination for hysteroscopic procedures yields effective analgesia, limited cardiopulmonary depression, and a low incidence of adverse events (Song et al., 2025). Nevertheless, optimal dosing regimens that incorporate multimodal nociceptive and sedation monitoring remain to be defined.

In particular, prior studies of propofol-oliceridine combinations have relied primarily on sedation depth indices such as the Bispectral Index (BIS), without incorporating dynamic nociceptive-antinociceptive balance monitoring tools like the Nociception Level Index (NOX). This gap may result in inaccurate dose estimation and suboptimal anesthetic management. Despite the widespread use of the BIS in clinical practice, we opted to utilize the Cerebral State Index (CSI) for the following reasons. First, CSI demonstrates comparable accuracy to BIS in assessing the depth of sedation during general anesthesia (Zhong et al., 2005), and exhibits greater resistance to electrosurgical interference (Nishiyama and Komatsu 2010)—a consideration of particular importance in hysteroscopic procedures that may involve electrocautery. Second, the DMT-101 monitor used in this study integrates CSI with the NOX monitoring on a single platform, enabling simultaneous assessment of sedation and analgesia. Third, CSI employs a fuzzy logic algorithm—specifically, an Adaptive Neuro-Fuzzy Inference System (ANFIS)—to integrate multiple electroencephalographic parameters (including the β-ratio, α-ratio, β−α ratio, and burst suppression ratio), converting them into a quantitative index scaled from 0 to 100, which demonstrates good correlation with clinical sedation scores (Hoymork et al., 2007). Collectively, these attributes—resistance to electrosurgical interference, integrated sedation–analgesia monitoring, and a robust algorithmic framework—rendered CSI particularly well suited for the hysteroscopic setting.

We therefore designed a two-phase dose-finding study using Dixon’s up-and-down sequential method. The primary objective was to determine the ED_50_ and ED_95_ of propofol coadministered with a fixed dose of oliceridine (Phase I), and then the ED_50_ and ED_95_ of oliceridine combined with the Phase I ED_50_ of propofol (Phase II). NOX and CSI were monitored concurrently to provide a more comprehensive assessment of analgesic and sedation status. We hypothesized that this integrated approach would yield practical dosing guidance to improve anesthetic outcomes in outpatient painless hysteroscopy.

## 2 Methods

### 2.1 Study design and patients

This prospective, non-randomized, single-arm study employed Dixon’s sequential dose-finding method. Ethical approval was obtained from the Ethics Committee of Shunde Hospital Affiliated to Jinan University (DSY-LL-2024174, 15 August 2024), and the study was registered with the Chinese Clinical Trial Registry (www.chictr.org.cn, ChiCTR2500102118, 8 May 2025). Patients were recruited from the Department of Gynecology at our institution. Before enrollment, written informed consent was secured from all participants. The conduct of this study adhered to institutional policies as well as the ethical frameworks established by the Declaration of Helsinki and CIOMS international guidelines.

### 2.2 Criteria for inclusion and exclusion

We recruited women aged 18–60 years, with ASA physical status I–II, BMI 18–28 kg/m^2^, and Mallampati classification I–II, scheduled for elective hysteroscopy under intravenous anesthesia. Exclusion criteria included: known allergy to propofol or oliceridine; upper respiratory infections, airway hyperresponsiveness, or anticipated difficult airway; severe cardiovascular, pulmonary, hepatic, or renal dysfunction; adrenal insufficiency; psychiatric disorders or hyperthyroidism; alcohol abuse within 24 h; or recent use of psychoactive/analgesic medications. The study included both diagnostic and operative (therapeutic) hysteroscopic procedures. However, procedures anticipated to exceed 30 min (e.g., hysteroscopic myomectomy, endometrial ablation) were excluded to minimize the confounding effects of prolonged drug metabolism, cumulative pharmacodynamic effects, and risks of fluid overload. The majority of enrolled patients underwent diagnostic hysteroscopy or brief operative procedures such as polypectomy.

### 2.3 Anesthesia procedure

Patients fasted for at least 6 h and abstained from clear fluids for at least 2 h before surgery. On arrival in the operating room, an intravenous cannula was placed on the dorsum of the patient’s hand. Standard monitoring included electrocardiography, SpO_2_, HR, and MAP. A DMT-101 monitor (Danmit Medical Technology Co., Ltd., China) was used for continuous NOX and CSI recording. Supplemental oxygen was supplied through a nasal cannula at a flow rate of 3 L/min. Baseline vital signs were recorded after a 3-min rest period. The study comprised two sequential phases. In Phase I, 1 mg of oliceridine fumarate (Jiangsu Enhua Pharmaceutical Co., Ltd., China; diluted to 0.1 mg/mL with normal saline; batch TAG25E02) was administered intravenously. Two minutes later, propofol (Fresenius Kabi AB, Austria; 0.2 g/20 mL; batch 10TL3020) was titrated using Dixon’s up-and-down method. The first patient received 2 mg/kg of propofol, and subsequent patients received doses adjusted in 0.1 mg/kg increments or decrements. Once clinical signs confirmed adequate sedation (loss of eyelash reflex, no response to verbal stimuli) and NOX and CSI reached stable plateaus for at least 30 s, hysteroscopy was initiated and the dose-response was assessed. An effective response led to a dose reduction for the next patient; an ineffective response led to a dose increase. This process continued until seven positive-negative crossover points were achieved (Pace and Stylianou, 2007). In Phase II, oliceridine was administered first, starting at 1 mg with 0.1 mg increments (Ni et al., 2024); 2 minutes later, the Phase I ED50 dose of propofol was given. The same dose-response assessment criteria as in Phase I were applied. Using Dixon’s sequential allocation method, the doses of oliceridine and propofol were titrated according to the response observed in the immediately preceding patient. When body movement, frowning, or a Ramsay Sedation Scale score <5 occurred during cervical dilation or hysteroscope insertion, the following rescue protocol was implemented: the procedure was temporarily halted, and a supplemental bolus of propofol (0.5 mg/kg) was administered intravenously. If the inadequate response persisted after 60 s, an additional dose of propofol (0.5 mg/kg) was given. The procedure was resumed only after adequate sedation was achieved (loss of eyelash reflex, no response to verbal stimuli, Ramsay Sedation Scale ≥5). All rescue events were recorded.

### 2.4 Outcome assessments

The primary endpoint was the effective dose required to suppress the stress response elicited by cervical dilation and hysteroscope insertion. For each patient, the administered dose was judged effective when it produced no body movements and no frowning, with a Ramsay Sedation Scale score ≥5 during cervical dilation and hysteroscope insertion, and ineffective when body movements, frowning, or a Ramsay Sedation Scale score <5 were observed during the same procedures (Chen et al., 2021).

Secondary outcomes included intraoperative monitoring parameters (HR, MAP, SpO_2_, and trends in NOX and CSI) recorded at five time points: entering the operating room (T0), 1 min post-induction (T1), first insertion of the hysteroscope (T2), end of the hysteroscopic procedure (T3), and consciousness recovery (T4). Additional secondary outcomes were the use of rescue measures (vasoactive drugs, supplemental analgesia, or airway support), adverse events (hypotension, bradycardia, SpO_2_ <93%, jaw thrust, injection pain, nausea, or vomiting), and postoperative uterine cramping pain assessed by VAS (0–10) in the recovery area. CSI values of 40–60 generally indicate appropriate surgical sedation (Lenkin et al., 2014), while NOX values of 25–50 reflect adequate analgesia (Ledowski, 2019).

Adverse events were handled as described below. When hypotension occurred, defined as MAP below 60 mmHg or a reduction exceeding 30% from baseline, intravenous ephedrine 6 mg was administered. Respiratory depression (SpO_2_ <90% for ≥15 s) was managed by increasing supplemental oxygen delivery via face mask and, if needed, positive pressure ventilation. SpO_2_ <93% at any time was recorded as an adverse event. Bradycardia (HR < 50 beats/min) was treated with intravenous atropine 0.5 mg. Postoperative pain (VAS >3) was treated with intravenous flurbiprofen axetil 50 mg.

### 2.5 Blinding method

Patients were enrolled sequentially based on admission order and scheduled procedure time. Dixon’s up-and-down dose-adjustment rules were predefined for both phases. To reduce allocation bias, dose sequence and adjustment rules were concealed from the clinical team until data collection was complete for each patient. Drug preparation was performed by nurses not involved in assessment or outcome recording. Anesthesiologists, surgeons, and postoperative care staff were blinded to the specific dose within the sequential protocol. Patients were also unaware of their dose levels.

### 2.6 Statistical analysis

Sample size was driven by Dixon’s sequential design, which requires seven effective-ineffective crossover points for stable dose-response estimation (approximately 20–30 patients per phase). Statistical analysis was conducted with SPSS (version 26.0) and Python (version 3.13). Normally distributed continuous variables were reported as mean ± SD and compared by Student’s t-test (two groups) or one-way ANOVA (multiple groups) after confirming variance homogeneity. Non-normal variables were expressed as median (IQR) and compared by Mann-Whitney U-test or Kruskal-Wallis H-test. Categorical variables were presented as n (%) and analyzed by chi-square or Fisher’s exact test. ED50 and ED95 were estimated from probit regression models, with 95% CIs calculated by the delta method. Model fit was assessed using the Pearson goodness-of-fit test. For sensitivity analysis, isotonic regression with the pool-adjacent-violators algorithm (PAVA) was applied; ED50 was estimated by linear interpolation on the fitted isotonic curve, and 95% CIs were obtained from 2,000 bootstrap resamples. Two-sided P < 0.05 was considered statistically significant.

## 3 Results

### 3.1 Patient information

Fifty-two gynecological patients were enrolled between 16 May 2025 and 23 October 2025. One participant withdrew preoperatively due to anxiety and was excluded, leaving 28 patients in Phase I and 23 in Phase II for final analysis (Figure 1). Dose-response sequences are shown in Figures 2, 3. In Phase I, 15 responses were effective and 13 ineffective; in Phase II, 14 were effective and nine ineffective. Baseline characteristics, encompassing ASA classification, age, height, weight, and BMI, revealed no significant intergroup differences (P > 0.05; Tables 1, 2). Notably, all patients in Phase II were ASA II, whereas Phase I included both ASA I and II patients.

**FIGURE 1:**
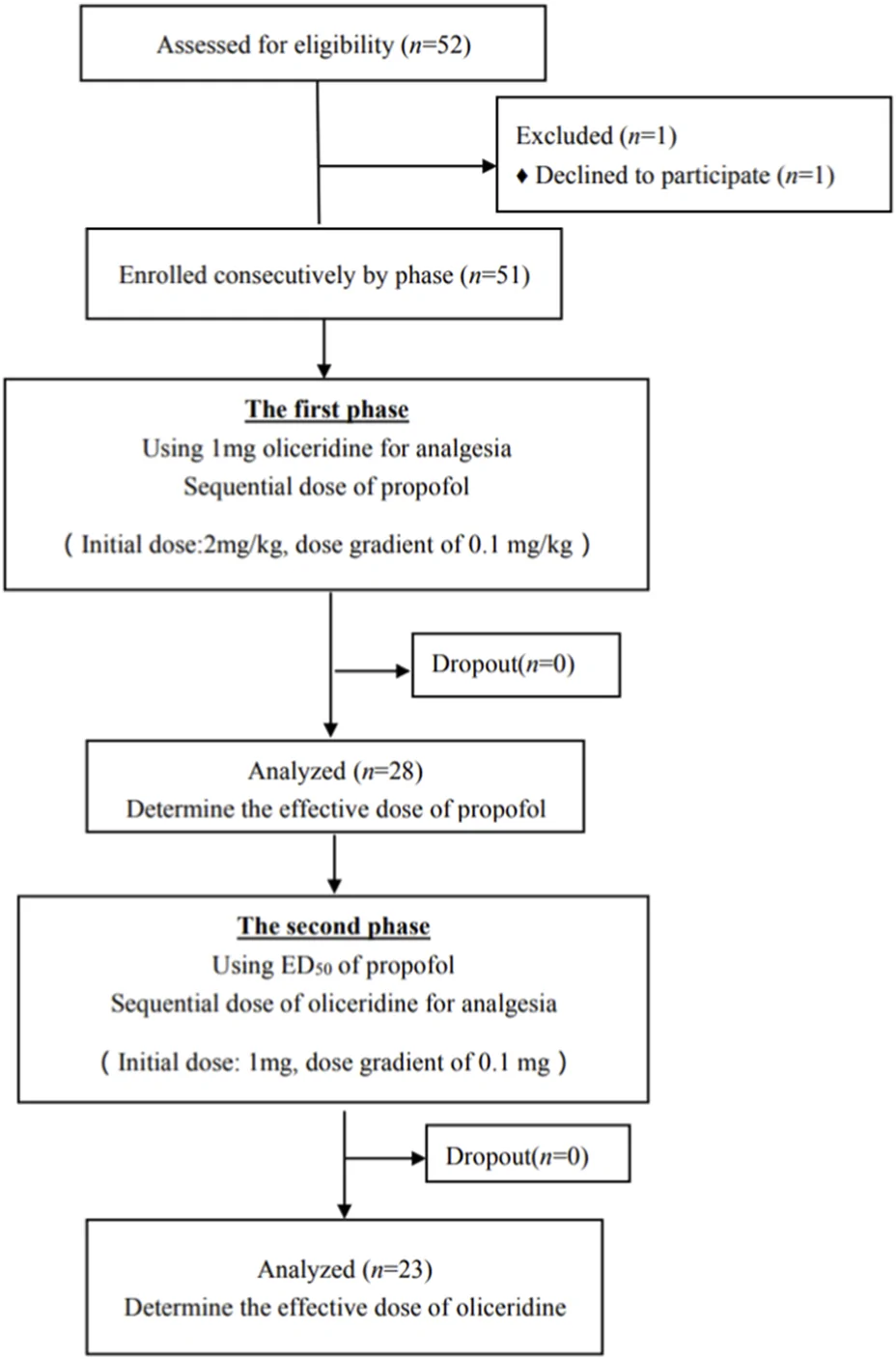
Flow diagram of the study.

**FIGURE 2:**
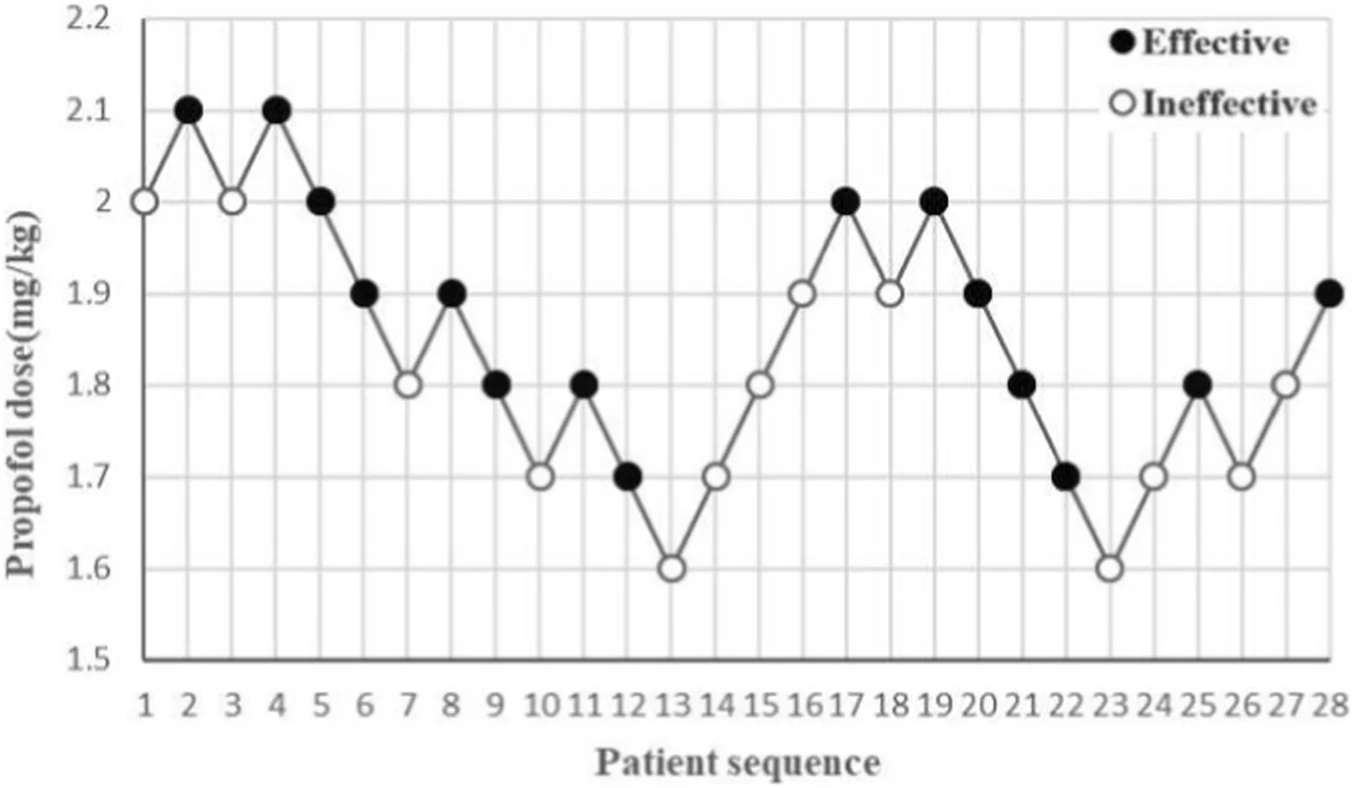
Sequential test of induced dose of propofol.

**FIGURE 3:**
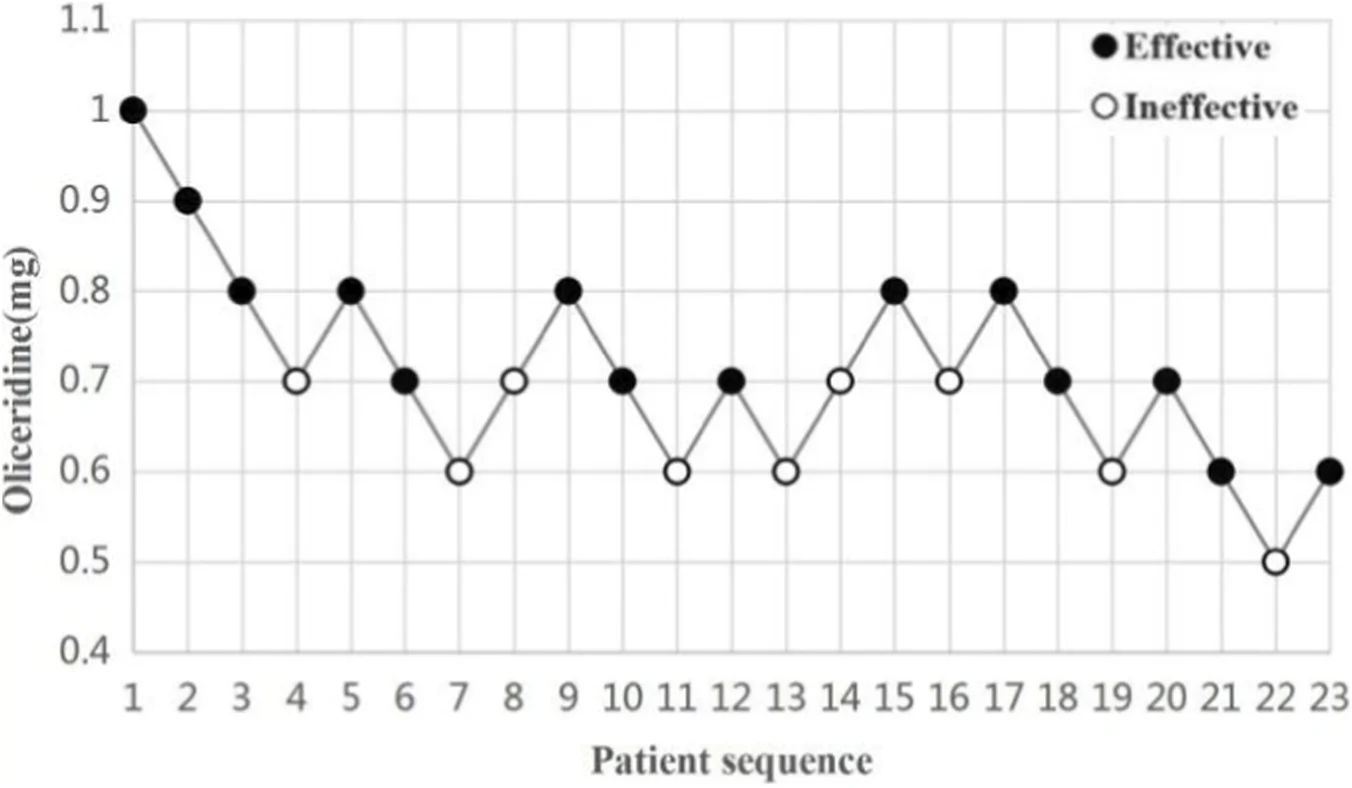
Sequential test of induced dose of oliceridine.

**TABLE 1:** Patients’ characteristics of the first phase.

Variable	Total (n = 28)	Effective (n = 15)	Ineffective (n = 13)	*P*
ASA (I/II)	7/21	4/11	3/10	1
Age (years)	41.1 ± 8.6	42.1 ± 6.8	39.9 ± 10.4	0.507
Height (cm)	156.3 ± 4.1	156.8 ± 4.7	155.6 ± 3.3	0.451
Weight (kg)	55.4 ± 6.9	55.4 ± 6.3	55.3 ± 7.8	0.994
BMI (kg/m^2^)	22.5 ± 2.6	22.5 ± 2.1	22.5 ± 3.2	0.972

Continuous variables are presented as mean ± SD, and categorical data as counts. Normally distributed continuous variables were compared using Student’s t-test; non-normally distributed data were analyzed with the Mann–Whitney U test; categorical data were compared using the χ^2^ test. Abbreviations: ASA, american society of anesthesiologists; BMI, body mass index.

**TABLE 2:** Patients’ characteristics of the second phase.

Variable	Total (n = 23)	Effective (n = 14)	Ineffective (n = 9)	*P*
ASA (I/II)	0/23	0/14	0/9	-
Age (years)	45.3 ± 7.7	45.9 ± 7.2	44.2 ± 8.8	0.618
Height (cm)	157.8 ± 5.2	157.9 ± 5.1	157.7 ± 5.5	0.934
Weight (kg)	59.7 ± 8.2	62.0 ± 8.2	55.9 ± 7.2	0.084
BMI (kg/m^2^)	23.8 ± 2.6	24.6 ± 2.5	22.6 ± 2.3	0.069

Continuous variables are presented as mean ± SD, and categorical data as counts. Normally distributed continuous variables were compared using Student’s t-test; non-normally distributed data were analyzed with the Mann–Whitney U test; categorical data were compared using the χ^2^ test. Abbreviations: ASA, american society of anesthesiologists; BMI, body mass index.

### 3.2 CSI and NOX values at T2

At T2 (Table 3), all patients with an effective response (Ramsay ≥5) had CSI ≤60 in both Phase I (100%) and Phase II (100%). Among these responders, NOX ≤50 was achieved in 73.3% (11/15) of Phase I and 64.3% (9/14) of Phase II patients. Patients with ineffective responses showed lower adherence: CSI ≤60 in 46.2% (6/13) and 66.7% (6/9) for Phases I and II, respectively, and NOX ≤50 in only 15.4% (2/13) of Phase I and 0% of Phase II cases.

**TABLE 3:** Changes in CSI and NOX during the T2 period.

Variable	The first phase		The second phase	
	Effective (n = 15)	Ineffective (n = 13)	Effective (n = 14)	Ineffective (n = 9)
CSI≤60, n (%)	15 (100)	6 (46.2)	14 (100)	6 (66.7)
NOX≤50, n (%)	11 (73.3)	2 (15.4)	9 (64.3)	0 (0)

Data are expressed as n [%].

Abbreviations: CSI, cerebral state index; NOX, nociception level index.

### 3.3 ED_50_ and ED_95_ of propofol and oliceridine

Probit regression yielded the following effective doses. In Phase I, the propofol ED_50_ was 1.818 mg/kg (95% CI: 1.696–1.940) and ED_95_ was 2.223 mg/kg (95% CI: 1.840–2.605). In Phase II, the oliceridine ED_50_ was 0.663 mg (95% CI: 0.605–0.720) and ED_95_ was 0.813 mg (95% CI: 0.692–0.934) (Figure 4). Pearson goodness-of-fit tests indicated adequate model fit (Phase I: χ^2^ = 1.897, df = 4, P = 0.755; Phase II: χ^2^ = 1.069, df = 4, P = 0.899). Isotonic regression was additionally performed as a sensitivity check. The resulting ED50 estimates—1.770 mg/kg (95% CI: 1.677–2.011) for propofol and 0.675 mg (95% CI: 0.580–0.733) for oliceridine—differed from the probit-derived values by no more than 3%, indicating that the primary estimates were robust (Table 4).

**FIGURE 4:**
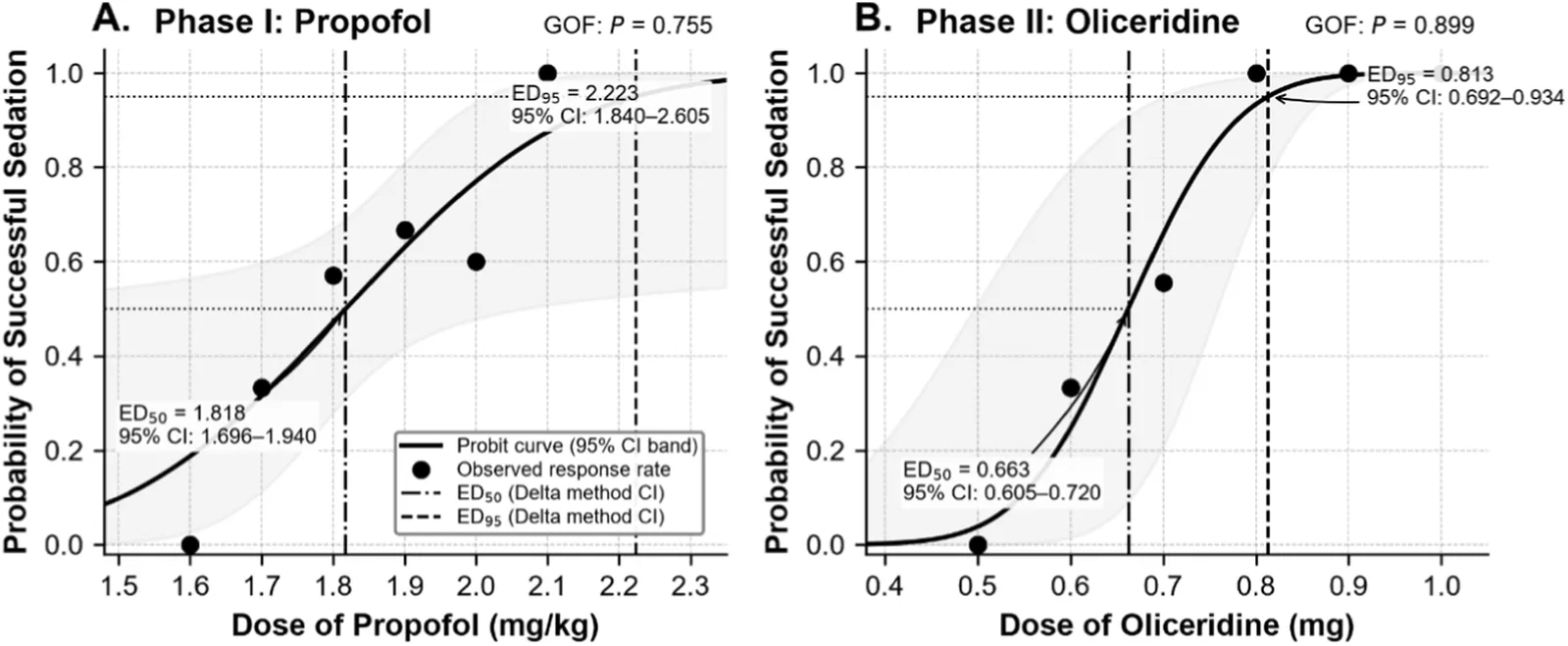
Probit dose-response curves from the two-phase up-and-down sequential study **(A)** Phase I: propofol dose titration with fixed oliceridine (1 mg), n = 28. **(B)** Phase II: oliceridine dose titration with propofol fixed at the Phase I ED_50_, n = 23. Solid curves represent fitted probit models; shaded bands denote 95% confidence intervals; circles indicate observed response rates at each dose level. Vertical dashed lines mark the estimated ED_50_ and ED_95_ with delta-method 95% confidence intervals (horizontal bars). GOF, goodness-of-fit P value.

**TABLE 4:** Comparison of three 95% confidence interval estimation methods for effective dose estimates (ED_50_ and ED_95_).

Parameter	Point estimate	Delta method 95% CI	Fieller method 95% CI	Profile likelihood 95% CI	GOF P
Phase I: propofol titrated (n = 28)					
Propofol ED50 (mg/kg)	1.818	1.696–1.940	1.296–2.063	1.598–1.994	0.755
Propofol ED95 (mg/kg)	2.223	1.840–2.605	2.015–8.305	2.017–2.803	
Phase II: oliceridine titrated (n = 23)					
Oliceridine ED50 (mg)	0.663	0.605–0.720	0.534–0.734	0.569–0.726	0.899
Oliceridine ED95 (mg)	0.813	0.692–0.934	0.739–1.363	0.732–1.021	

Delta method: linear approximation of the variance; recommended for reporting in this study. Fieller method: exact CI based on Fieller’s theorem; produces extremely wide CIs when g1(Phase I: g = 0.884). Profile likelihood: based on the likelihood ratio statistic; provides intermediate CI width.

### 3.4 Hemodynamic changes

Hemodynamic data are summarized in Table 5. In Phase I, HR at T3 was significantly lower than baseline (T0) (P < 0.05). MAP differed significantly from baseline at T1, T2, and T3 in Phase I (all P < 0.05), and at T1, T2, T3, and T4 in Phase II (all P < 0.05). Between-phase comparisons showed no significant differences in MAP at any time point, except for HR at T3.

**TABLE 5:** Changes in hemodynamics during the study.

Index	Time points	The first phase	The second phase
HR (bpm)	T0 T1 T2 T3 T4	79.4 ± 16.3 75.6 ± 11.5 73.9 ± 10.1 67.0 ± 9.4* 73.4 ± 11.4	78.1 ± 14.6 78.4 ± 10.7 75.3 ± 9.5 73.0 ± 10.4# 75.1 ± 11.5
MAP (mmHg)	T0 T1 T2 T3 T4	98.9 ± 11.6 77.8 ± 11.3* 79.0 ± 10.9* 91.9 ± 10.8* 93.8 ± 9.0	103.5 ± 14.2 82.3 ± 12.7* 82.6 ± 10.1* 91.8 ± 13.2* 93.0 ± 15.1*

T0: entering the operating room; T1: 1 min post-induction; T2: at the first insertion of hysteroscopy; T3: at the end of hysteroscopy operation; T4: At the time of consciousness recovery. *p: Significantly different from T0 (p < 0.05).

^#^ *p*: Significantly different from T1 (p < 0.05).

Abbreviations: HR, heart rate; MAP, mean arterial pressure.

### 3.5 Adverse events

There were no significant differences in anesthesia-related adverse events between phases (P > 0.05; Table 6). Fisher’s exact test was used for all comparisons due to small expected cell counts. These events included hypotension, bradycardia, SpO_2_ <93%, jaw thrust, injection pain, nausea, vomiting, and postoperative uterine cramping pain.

**TABLE 6:** Anesthesia-related adverse events.

Variable	The first phase n = 28	The second phase n = 23	*P*
Hypotension, n (%)	4 (14.3)	3 (13.0)	1
Bradycardia, n (%)	2 (7.1)	1 (4.3)	1
SpO_2_ <93%, n (%)	4 (14.3)	2 (8.6)	1
Postoperative uterine cramping pain, VAS≥3, n (%)	2 (7.1)	1 (4.3)	1
Nausea and vomiting, n (%)	0 (0)	0 (0)	-
Jaw thrust, n (%)	8 (28.6)	4 (17.4)	0.349
Injection pain, VAS≥3, n (%)	2 (7.1)	4 (17.4)	0.488
Intubation, n (%)	0 (0)	0 (0)	-
Dizziness, n (%)	7 (25.0)	1 (4.3)	0.103

Data are expressed as n [%]. A chi-square test was used to assess differences of adverse events.

Abbreviations: SpO_2_, peripheral oxygen saturation; VAS, visual analogue scale.

## 4 Discussion

The study was designed in two phases to identify the effective doses of propofol and oliceridine for painless hysteroscopy. Dixon’s up-and-down method served as the core dose-finding approach, supplemented by simultaneous NOX/CSI monitoring throughout the procedure. Phase I yielded a propofol ED_50_ of 1.818 mg/kg (95% CI: 1.696–1.940) and ED_95_ of 2.223 mg/kg (95% CI: 1.840–2.605) when combined with 1 mg oliceridine. Phase II, in which propofol was fixed at the Phase I ED_50_, produced an oliceridine ED_50_ of 0.663 mg (95% CI: 0.605–0.720) and ED_95_ of 0.813 mg (95% CI: 0.692–0.934). These estimates offer preliminary dosing references for women aged 18–60 years, though clinical use should consider individual patient variability.

We chose to fix propofol at the Phase I ED_50_ (rather than the ED_95_) in Phase II for the following reasons: First, using the ED_50_ as the fixed background dose allows the dose-response relationship of oliceridine to be characterized around the midpoint of the propofol dose-response curve, maximizing the sensitivity of the sequential allocation design to detect effective and ineffective responses. Second, fixing propofol at the ED_95_ would have likely resulted in an unacceptably high rate of effective responses in Phase II, providing insufficient crossover points for reliable oliceridine dose estimation. Third, from a safety perspective, using the ED_50_ of propofol (1.818 mg/kg) minimizes the risk of excessive sedation and respiratory depression when combined with a titrated opioid, consistent with the principle of dose-sparing in combination therapy. We acknowledge that in routine clinical practice, anesthesiologists may prefer the ED_95_ to ensure a more reliable anesthetic effect; however, the study design required the ED_50_ to generate informative crossover data for oliceridine dose estimation.

Propofol, a GABA_A receptor agonist, and oliceridine, a μ-opioid receptor biased agonist, act through complementary mechanisms. This pharmacodynamic synergy likely explains why the propofol ED_50_ in our combination (1.818 mg/kg) was was lower than the 1.985 mg/kg reported with propofol-sufentanil combinations (Jin et al., 2024), and permitted dose reduction of both agents, which may help decrease respiratory depression and other opioid-related adverse effects. However, because this was a single-arm study without a comparator group, we cannot determine whether the propofol-oliceridine regimen is superior to propofol alone or to combinations such as propofol-sufentanil, propofol-remifentanil, or propofol-fentanyl. Similarly, the lower propofol ED_50_ observed in our combination should not be interpreted as evidence of reduced respiratory depression compared with conventional regimens, as no direct comparison was performed. Comparative studies with appropriate control groups are needed to evaluate the relative safety and efficacy of the propofol-oliceridine combination.

Safety outcomes warrant cautious interpretation. The need for jaw thrust in 28.6% of Phase I and 17.4% of Phase II patients indicates a clinically relevant degree of upper airway obstruction and respiratory depression associated with this regimen. These rates are non-trivial and underscore the importance of continuous airway monitoring and readiness for airway intervention when using the propofol-oliceridine combination. The occurrence of SpO_2_ <93% in 14.3% (Phase I) and 8.6% (Phase II) of patients further supports the presence of measurable respiratory depressant effects. We therefore caution against interpreting these findings as evidence of an absence of clinically relevant respiratory effects. That said, other aspects of the safety profile were more favorable. The low rate of postoperative nausea and vomiting aligns with prior findings (Soergel et al., 2014a) and is consistent with oliceridine’s G-protein-biased mechanism, which activates analgesic pathways while minimizing β-arrestin-mediated adverse effects (Bergese et al., 2020; Moss et al., 2023; Singla et al., 2019; Urits et al., 2019). Oliceridine is primarily metabolized in the liver into inactive products and subsequently cleared via both bile and urine. This pharmacokinetic profile minimizes accumulation potential, making the drug appropriate for individuals with mild to moderate kidney or liver dysfunction (Oliceridine, 2012). Its rapid onset, favorable side-effect profile, and lower abuse potential make it well suited for ambulatory anesthesia (James and Jowza, 2019; Niu et al., 2023).

Several methodological points deserve discussion. We used the delta method to compute 95% CIs for ED50 and ED95, as it produces symmetric, clinically interpretable intervals through local linear approximation of the probit transformation. The delta method CIs (e.g., propofol ED95: 1.840–2.605 mg/kg) were considerably narrower than Fieller’s theorem intervals (2.015–8.305 mg/kg). This difference stems from the Fieller g-statistic (g = 0.884 for Phase I), which approaches 1 when the slope parameter is only marginally significant, producing extremely wide, asymmetric intervals. The Fieller upper bound of 8.305 mg/kg for propofol ED95, for example, far exceeds any clinically relevant dose. The delta method yielded intervals that were both statistically defensible and clinically plausible, and was therefore selected as the primary reporting method. Agreement between probit-derived and isotonic regression-derived ED50 estimates (1.818 vs. 1.770 mg/kg for propofol; 0.663 vs. 0.675 mg for oliceridine; deviations <3%) supports the internal validity of our results. Isotonic regression, being non-parametric and free of distributional assumptions, is particularly valuable as a sensitivity check in small-sample studies (Vågerö and Sundberg, 1999; Görges et al., 2017). The 0.1 mg/kg dose step for propofol, while clinically practical, may limit resolution at distribution tails, contributing to uncertainty in ED95 estimates (Oron, Souter, and Flournoy, 2022). This limitation highlights the need for individualized titration, as age and BMI can substantially affect dose requirements (Zhu et al., 2025; Zhao et al., 2025). The biased coin design, an alternative sequential method, could improve statistical efficiency but adds operational complexity (George et al., 2010). Clinicians should therefore balance the practical advantages of Dixon’s method against its limitations when applying these dose estimates.

Importantly, the Dixon up-and-down sequential method was originally designed to estimate the median effective dose (ED_50_), not the ED_95_. Estimation of ED_95_ under a small-sample sequential allocation design is associated with considerable statistical uncertainty, as reflected by the wide confidence intervals observed in our study (e.g., propofol ED_95_ 95% CI: 1.840–2.605 mg/kg). The ED_95_ estimates should therefore be interpreted as preliminary and indicative rather than definitive. Larger studies employing biased coin designs or concentrated dose-response assessments at the upper tail of the dose-response curve would be needed to obtain more precise ED_95_ estimates. The 0.1 mg/kg dose increment for propofol was selected based on several considerations: (1) This increment is consistent with the dose step sizes used in prior Dixon up-and-down studies of propofol for procedural sedation (Chen et al., 2021; Yu et al., 2019); (2) It represents approximately 5% of the anticipated ED_50_ range, which provides adequate resolution for dose-response estimation while remaining clinically practical (3) Smaller increments (e.g., 0.05 mg/kg); would have required a substantially larger sample size to achieve the same number of crossover points, which was not feasible for a single-center exploratory study. The 0.1 mg dose increment for oliceridine was selected based on the pharmacokinetic properties of the drug and prior dose-finding studies (Song et al., 2025).

A notable feature of our study is the simultaneous use of NOX and CSI to monitor analgesia and sedation, an approach not widely adopted in prior hysteroscopy research (Gao et al., 2024). NOX offers real-time assessment of nociceptive-antinociceptive balance, guiding opioid and sedative titration (Luginbühl et al., 2010). The low rate of postoperative uterine cramping in our cohort may reflect both oliceridine’s analgesic efficacy and the precision of NOX-guided dosing. Recent studies confirm that NOX can reliably evaluate intraoperative analgesia and may help reduce postoperative delirium (Du et al., 2025). Furthermore, in our data, a Ramsay score ≥5 consistently corresponded to CSI ≤60, which also supports the view that CSI serves as a reliable tool for preventing both intraoperative awareness and oversedation, with performance comparable to BIS (González-Mendibil et al., 2024).

However, we acknowledge that no formal correlation analysis between NOX/CSI values and clinical efficacy outcomes was performed. Future studies should incorporate such analyses to validate the predictive value of these monitoring modalities. The absence of this correlation analysis is acknowledged as a limitation of the present study. The observed mismatch between clinical effectiveness and NOX values (26.7%–35.7% of effective responders had NOX >50) warrants further discussion. This discrepancy may arise from several factors: (1) The NOX algorithm may not fully account for the unique pharmacodynamic profile of oliceridine as a biased μ-opioid receptor agonist; (2) Clinical signs of effectiveness (absence of body movement) may reflect the effects of propofol-induced immobility rather than true analgesia; (3) The NOX threshold of 50 was derived from studies using traditional opioids and may not be directly transferable to oliceridine-based regimens. These observations suggest that NOX thresholds may need to be recalibrated for novel opioid agents, and that combining objective monitoring with clinical assessment remains essential.

Several limitations should be noted. First, enrollment was limited to women aged 18–60 years with ASA I–II status. Elderly patients and those at body weight extremes were not included, and since age and body composition can affect drug pharmacokinetics, our ED_50_ and ED_95_ values may not generalize beyond relatively healthy adult women. Second, obstetric history—including differences in cervical compliance between nulliparous and parous women—was not analyzed as a covariate, though such anatomical variation may influence procedural stimulation and analgesic requirements (Chaves et al., 2021). Third, the single-center design may limit external generalizability. Further multi-center studies involving diverse, larger cohorts and detailed subgroup analyses are necessary to establish the generalizability of these outcomes. Finally, as noted above, the delta, Fieller, and profile-likelihood methods can produce materially different CI estimates for ED95 in small samples. Larger studies would help reduce this sensitivity and yield more precise tail estimates. Additionally, because the majority of enrolled patients underwent diagnostic or brief operative hysteroscopy, the estimated ED_50_ and ED_95_ values are best interpreted as single-bolus dose requirements under the specific conditions of this study. As the sequential up-and-down method determines the dose–response relationship from a single predetermined dose administered to each patient, procedure duration *per se* does not confound these estimates; a prolonged procedure would primarily increase total drug consumption (e.g., through additional boluses or continuous infusion) rather than alter the dose–response relationship itself. Nevertheless, extrapolation of these single-dose estimates to prolonged therapeutic hysteroscopic procedures—which typically necessitate cumulative or maintenance dosing and carry a higher risk of fluid overload—should be made with caution, and the dose requirements for such procedures warrant dedicated evaluation in future studies. Furthermore, although NOX and CSI were monitored concurrently, no formal correlation analysis between these indices and clinical efficacy outcomes was performed, which precludes definitive conclusions about their predictive value for clinical effectiveness. Future studies should incorporate such analyses to validate the predictive value of these monitoring modalities.

This study determined the ED50 and ED95 values for the propofol-oliceridine combination in painless hysteroscopy, providing preliminary dosing guidance for clinical practice. This drug pairing demonstrated a favorable safety and efficacy profile. Multi-institutional studies with larger cohorts are warranted to confirm these results and broaden their clinical applicability.

## Data Availability

The original contributions presented in the study are included in the article/supplementary material, further inquiries can be directed to the corresponding author.

## References

[B1] Bergese S. D. Brzezinski M. Hammer G. B. Beard T. L. Pan P. H. Mace S. E. (2019). ATHENA: a phase 3, open-label Study of the safety and effectiveness of oliceridine (TRV130), A G-Protein selective agonist at the µ-Opioid receptor, in patients with moderate to severe acute pain requiring parenteral opioid therapy. J. Pain Res. 12, 3113–3126. 10.2147/jpr.S217563 31814753 PMC6861532

[B2] Bergese S. Berkowitz R. Rider P. Ladouceur M. Griffith S. Segura Vasi A. (2020). Low incidence of postoperative respiratory depression with oliceridine compared to morphine: a retrospective chart analysis. Pain Res. Manag. 2020, 7492865. 10.1155/2020/7492865 33163127 PMC7604609

[B3] Chaves I. A. Baêta T. Dolabella G. B. Barbosa L. R. Almeida N. M. Oliveira F. R. (2021). Pain scores at the insertion of the 52 MG levonorgestrel-releasing intrauterine system among nulligravidas and parous women. Eur. J. Contracept. Reprod. Health Care 26 (5), 399–403. 10.1080/13625187.2021.1925882 34096433

[B4] Chen C. Tang W. Ye W. Zhong W. Li Y. (2021). ED50 of propofol combined with nalbuphine on the sedative effect in painless hysteroscopy. Pain Ther. 10 (2), 1235–1243. 10.1007/s40122-021-00280-x 34131886 PMC8586435

[B5] Du Y. Cao J. Gao C. He K. Wang S. (2025). Influence of intraoperative pain management on postoperative delirium in elderly patients: a prospective single-center randomized controlled trial. Pain Ther. 14 (1), 387–400. 10.1007/s40122-024-00702-6 39757288 PMC11751207

[B6] Gao F. Xie Y. Zhu H. Chen C. Fu H. (2024). To compare the anesthetic effect of Remimazolam and propofol in painless hysteroscopic minimally invasive surgery. Ann. Ital. Chir. 95 (2), 159–165. 10.62713/aic.3291 38684498

[B7] George R. B. McKeen D. Columb M. O. Habib A. S. (2010). Up-down determination of the 90% effective dose of phenylephrine for the treatment of spinal anesthesia-induced hypotension in parturients undergoing cesarean delivery. Anesth. Analg. 110 (1), 154–158. 10.1213/ANE.0b013e3181c30b72 19910625

[B8] González-Mendibil I. García-Pascual E. Villanueva A. García-Gutiérrez S. (2024). Bispectral index monitoring for sedation in scheduled adult colonoscopy: a randomized controlled trial. Rev. Esp. Anestesiol. Reanim. Engl. Ed. 71 (9), 633–644. 10.1016/j.redare.2024.04.011 38663652

[B9] Görges M. Zhou G. Brant R. Ansermino J. M. (2017). Sequential allocation trial design in anesthesia: an introduction to methods, modeling, and clinical applications. Paediatr. Anaesth. 27 (3), 240–247. 10.1111/pan.13088 28211193

[B10] Goudra B. (2022). Oliceridine- opioid of the 21(st) century. Saudi J. Anaesth. 16 (1), 69–75. 10.4103/sja.sja_510_21 35261592 PMC8846232

[B11] Guraslan H. Senturk M. B. Dogan K. Yuksel B. Kaya C. Karacan T. (2022). Diagnostic office hysteroscopy; why is it still painful procedure despite the surgical experience and mini-hysteroscope? J. Obstet. Gynaecol. Res. 48 (6), 1418–1425. 10.1111/jog.15219 35274418

[B12] Hoymork S. C. Hval K. Jensen E. W. Raeder J. (2007). Can the cerebral state monitor replace the bispectral index in monitoring hypnotic effect during propofol/remifentanil anaesthesia? Acta Anaesthesiol. Scand. 51 (2), 210–216. 10.1111/j.1399-6576.2006.01213.x 17330331

[B13] Jalota L. Kalira V. George E. Shi Y. Y. Hornuss C. Radke O. (2011). Prevention of pain on injection of propofol: systematic review and meta-analysis. Bmj 342, d1110. 10.1136/bmj.d1110 21406529

[B14] James D. L. Jowza M. (2019). Treating opioid dependence: pain medicine physiology of tolerance and addiction. Clin. Obstet. Gynecol. 62 (1), 87–97. 10.1097/grf.0000000000000422 30614846

[B15] Jin L. Jiao C. C. Chen X. P. Sun L. H. Zhang Y. Cheng X. Z. (2024). The potency-ratio of ciprofol and propofol under procedural sedation and anesthesia for outpatient hysteroscopy during cervical dilation: a study using up-and-down sequential allocation method. BMC Anesthesiol. 24 (1), 426. 10.1186/s12871-024-02793-2 39592954 PMC11590266

[B16] Ledowski T. (2019). Objective monitoring of nociception: a review of current commercial solutions. Br. J. Anaesth. 123 (2), e312–e321. 10.1016/j.bja.2019.03.024 31047645 PMC6676047

[B17] Lenkin A. I. Zaharov V. I. Lenkin P. I. Smetkin A. A. Bjertnaes L. J. Kirov M. Y. (2014). Monitoring of anesthetic depth during surgical correction of acquired valvular disorders: single center, randomized trial. J. Cardiothorac. Vasc. Anesth. 28 (2), 301–307. 10.1053/j.jvca.2013.05.032 24094565

[B18] Luginbühl M. Schumacher P. M. Vuilleumier P. Vereecke H. Heyse B. Bouillon T. W. (2010). Noxious stimulation response index: a novel anesthetic state index based on hypnotic-opioid interaction. Anesthesiology 112 (4), 872–880. 10.1097/ALN.0b013e3181d40368 20216387

[B19] Ma B. Li Y. Leng C. Ji A. Zhang N. Tao X. (2025). A comparative evaluation of the safety and efficacy of oliceridine and sufentanil in gastrointestinal endoscopy: a Single-Center, randomized controlled trial. Drug Des. Devel Ther. 19, 5111–5121. 10.2147/dddt.S512529 40546662 PMC12182078

[B20] Moss L. Hijma H. Demitrack M. Kim J. Groeneveld G. J. van Velzen M. (2023). Neurocognitive effect of biased µ-Opioid receptor agonist oliceridine, a utility function analysis and comparison with morphine. Anesthesiology 139 (6), 746–756. 10.1097/aln.0000000000004758 37656771

[B21] Ni Y. Huang R. Yang S. Yang X. Y. Zeng S. Yao A. (2024). Pharmacokinetics and safety of oliceridine fumarate injection in Chinese patients with chronic non-cancer pain: a phase I, Single-Ascending-Dose, open-label clinical trial. Drug Des. Devel Ther. 18, 2729–2743. 10.2147/dddt.S461416 38974123 PMC11227858

[B22] Nishiyama T. Komatsu K. (2010). Cerebral state index *versus* bispectral index during propofol-fentanyl-nitrous oxide anesthesia. J. Anesth. 24 (3), 380–385. 10.1007/s00540-010-0906-5 20339885

[B23] Niu J. Hu W. Lu Y. Tang H. (2023). Efficacy and safety of oliceridine treatment in patients with postoperative pain: a systematic review and meta-analysis of randomized controlled trials. Expert Rev. Clin. Pharmacol. 16 (6), 589–599. 10.1080/17512433.2023.2213889 37171148

[B24] Nafziger A. N. Arscott K. A. Cochrane K. Skobieranda F. Burt D. A. Fossler M. J. (2020). The influence of renal or hepatic impairment on the pharmacokinetics, safety, and tolerability of oliceridine. Clin Pharmacol Drug Dev 9 (5), 639–650. 10.1002/cpdd.750 31697049 PMC7383509

[B25] Oron A. P. Souter M. J. Flournoy N. (2022). Understanding research methods: Up-and-down designs for dose-finding. Anesthesiology 137 (2), 137–150. 10.1097/aln.0000000000004282 35819863

[B26] Pace N. L. Stylianou M. P. (2007). Advances in and limitations of up-and-down methodology: a précis of clinical use, study design, and dose estimation in anesthesia research. Anesthesiology 107 (1), 144–152. 10.1097/01.anes.0000267514.42592.2a 17585226

[B27] Paulo A. A. S. Solheiro M. H. R. Paulo C. O. S. Afreixo V. M. A. (2016). What proportion of women refers moderate to severe pain during office hysteroscopy with a mini-hysteroscope? A systematic review and meta-analysis. Arch. Gynecol. Obstet. 293 (1), 37–46. 10.1007/s00404-015-3836-5 26253336

[B28] Ryu J. H. Kim J. H. Park K. S. Do S. H. (2008). Remifentanil-propofol *versus* fentanyl-propofol for monitored anesthesia care during hysteroscopy. J. Clin. Anesth. 20 (5), 328–332. 10.1016/j.jclinane.2007.12.015 18761238

[B29] Singla N. K. Skobieranda F. Soergel D. G. Salamea M. Burt D. A. Demitrack M. A. (2019). APOLLO-2: a randomized, placebo and active-controlled phase III Study investigating oliceridine (TRV130), a G protein-biased ligand at the μ-Opioid receptor, for management of moderate to severe acute pain following abdominoplasty. Pain Pract. 19 (7), 715–731. 10.1111/papr.12801 31162798 PMC6851842

[B30] Soergel D. G. Subach R. A. Burnham N. Lark M. W. James I. E. Sadler B. M. (2014a). Biased agonism of the μ-opioid receptor by TRV130 increases analgesia and reduces on-target adverse effects *versus* morphine: a randomized, double-blind, placebo-controlled, crossover study in healthy volunteers. Pain 155 (9), 1829–1835. 10.1016/j.pain.2014.06.011 24954166

[B31] Soergel D. G. Subach R. A. Sadler B. Connell J. Marion A. S. Cowan C. L. (2014b). First clinical experience with TRV130: pharmacokinetics and pharmacodynamics in healthy volunteers. J. Clin. Pharmacol. 54 (3), 351–357. 10.1002/jcph.207 24122908

[B32] Song D. Hamza M. White P. F. Klein K. Recart A. Khodaparast O. (2004). The pharmacodynamic effects of a lower-lipid emulsion of propofol: a comparison with the standard propofol emulsion. Anesth. Analg. 98 (3), 687–691. 10.1213/01.ane.0000103184.36451.d7 14980920

[B33] Song C. Z. Xu Y. Mei Z. Yao S. J. Song S. B. Guo L. (2025). Determination of the 90% effective dose of oliceridine combined with propofol for day-case hysteroscopy. Drug Des. Devel Ther. 19, 9343–9351. 10.2147/dddt.S546489 41116807 PMC12535727

[B34] Urits I. Viswanath O. Orhurhu V. Gress K. Charipova K. Kaye A. D. (2019). The utilization of Mu-Opioid receptor biased agonists: Oliceridine, an opioid analgesic with reduced adverse effects. Curr. Pain Headache Rep. 23 (5), 31. 10.1007/s11916-019-0773-1 30880365

[B35] Vågerö M. Sundberg R. (1999). The distribution of the maximum likelihood estimator in up-and-down experiments for quantal dose-response data. J. Biopharm. Stat. 9 (3), 499–519. 10.1081/bip-100101190 10473034

[B36] Yu J. Xiang B. Song Y. Chen H. Li Y. Liu C. (2019). ED50 of propofol in combination with low-dose sufentanil for intravenous anaesthesia in hysteroscopy. Basic Clin. Pharmacol. Toxicol. 125 (5), 460–465. 10.1111/bcpt.13280 31231918

[B37] Zhao J. Zhang Y. Su G. Wang S. Zhang X. Wang G. (2025). The median effective dose of ciprofol combined with a low-dose sufentanil for gastroscopy in obese or nonobese patients: a dose-finding study using Dixon's up-and-down method. Front. Pharmacol. 16, 1521715. 10.3389/fphar.2025.1521715 40041487 PMC11876871

[B38] Zhong T. Guo Q. L. Pang Y. D. Peng L. F. Li C. L. (2005). Comparative evaluation of the cerebral state index and the bispectral index during target-controlled infusion of propofol. Br. J. Anaesth. 95 (6), 798–802. 10.1093/bja/aei253 16210310

[B39] Zhu Q. Cui L. Li X. Li Q. Wang Y. (2025). Young, healthy, underweight women require higher effective doses of propofol for successful gastroscope insertion: a dose-finding Study using Dixon's up-and-down method. Drug Des. Devel Ther. 19, 3017–3028. 10.2147/dddt.S512554 40270991 PMC12015738

